# Extended Newick: it is time for a standard representation of phylogenetic networks

**DOI:** 10.1186/1471-2105-9-532

**Published:** 2008-12-15

**Authors:** Gabriel Cardona, Francesc Rosselló, Gabriel Valiente

**Affiliations:** 1 Department of Mathematics and Computer Science, University of the Balearic Islands, E-07122 Palma de Mallorca, Spain; 2 Algorithms, Bioinformatics, Complexity and Formal Methods Research Group, Technical University of Catalonia, E-08034 Barcelona, Spain

## Abstract

**Background:**

Phylogenetic trees resulting from molecular phylogenetic analysis are available in Newick format from specialized databases but when it comes to phylogenetic networks, which provide an explicit representation of reticulate evolutionary events such as recombination, hybridization or lateral gene transfer, the lack of a standard format for their representation has hindered the publication of explicit phylogenetic networks in the specialized literature and their incorporation in specialized databases. Two different proposals to represent phylogenetic networks exist: as a single Newick string (where each hybrid node is splitted once for each parent) or as a set of Newick strings (one for each hybrid node plus another one for the phylogenetic network).

**Results:**

The standard we advocate as extended Newick format describes a whole phylogenetic network with *k *hybrid nodes as a single Newick string with *k *repeated nodes, and this representation is unique once the phylogenetic network is drawn or the ordering among children nodes is fixed. The extended Newick format facilitates phylogenetic data sharing and exchange, and also allows for the practical use of phylogenetic networks in computer programs and scripts. This standard has been recently agreed upon by a number of computational biologists, is already supported by several phylogenetic tools, and avoids the different drawbacks of using an a priori unknown number of Newick strings without any additional mark-up to represent a phylogenetic network.

**Conclusion:**

The adoption of the extended Newick format as a standard for the representation of phylogenetic network is an important step towards the publication of explicit phylogenetic networks in peer-reviewed journals and their incorporation in a future database of published phylogenetic networks.

## Background

Phylogenetic networks provide an *explicit *representation of the evolutionary relationships among sequences, genes, chromosomes, genomes, or species. They differ from phylogenetic trees by the explicit modeling, by means of *hybrid nodes *instead of only *tree nodes*, of reticulate evolutionary events such as recombination, hybridization, or lateral gene transfer, and differ also from the *implicit *networks that allow for visualization and analysis of incompatible phylogenetic signals [1]. Phylogenetic networks have been extensively used in evolutionary studies, especially at the population level, where reticulate evolutionary events are quite common [2].

Over the past two decades, plant and animal biologists have been performing molecular phylogenetic analyses and submitting phylogenetic trees and associated data matrices to TreeBASE, a repository of phylogenetic trees [3], and some journals either require or encourage authors to submit their phylogenetic data to databases such as TreeBASE [4,5]. Sharing phylogenetic data has been eased by the multiple efforts to maintain such a database of published phylogenetic trees, which already contains over 5,000 phylogenetic trees with about 100,000 taxa from about 2,000 studies made by over 3,000 authors, but also by the adoption of a standard format for representing phylogenetic trees, the *Newick format*.

The Newick format [6,7], adopted June 26, 1986 by an informal committee meeting at Newick's seafood restaurant in Dover, New Hampshire, USA during the Society for the Study of Evolution meeting in Durham, New Hampshire, is the *de facto *standard for representing phylogenetic trees, and it is quite convenient since it makes it possible to describe a whole phylogenetic tree in linear form in a unique way, once the phylogenetic tree is drawn or the ordering among children nodes is fixed. The Newick description of a phylogenetic tree is a string of nested parentheses annotated with taxa names and possibly also with branch lengths or bootstrap values, obtained by traversing the phylogenetic tree in postorder and following some simple rules that allow for *parsing *a Newick string into the corresponding phylogenetic tree, and vice versa. In fact, almost every phylogenetic software tool includes an option to export phylogenetic trees in Newick format, and open-source code for parsing Newick strings is readily available in a number of software toolkits, including for instance BioPerl, BioPython, BioJava, and BioRuby.

When it comes to phylogenetic networks, however, there is not yet, to the best of our knowledge, any effort to build and maintain a database of published phylogenetic networks, nor do journals require or even encourage authors to submit explicit phylogenetic networks as supplementary material. The adoption of a standard representation for phylogenetic networks would certainly be an important first step towards reverting this situation. Every evolutionary biologist might certainly benefit from such a standard, because of the importance of standards for sharing data [8,9], and every computational biologist might also benefit as well, given the importance of standards for tool development [10].

**Figure 1:**
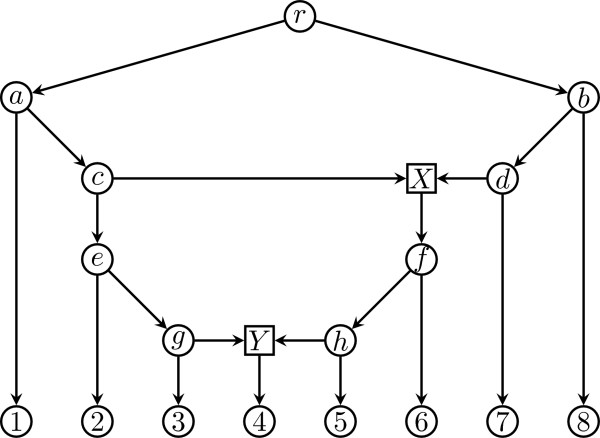
**A phylogenetic network**. A phylogenetic network *N*. Tree nodes are depicted as circles, and hybrid nodes as squares. Terminal nodes are numbered.

**Figure 2:**
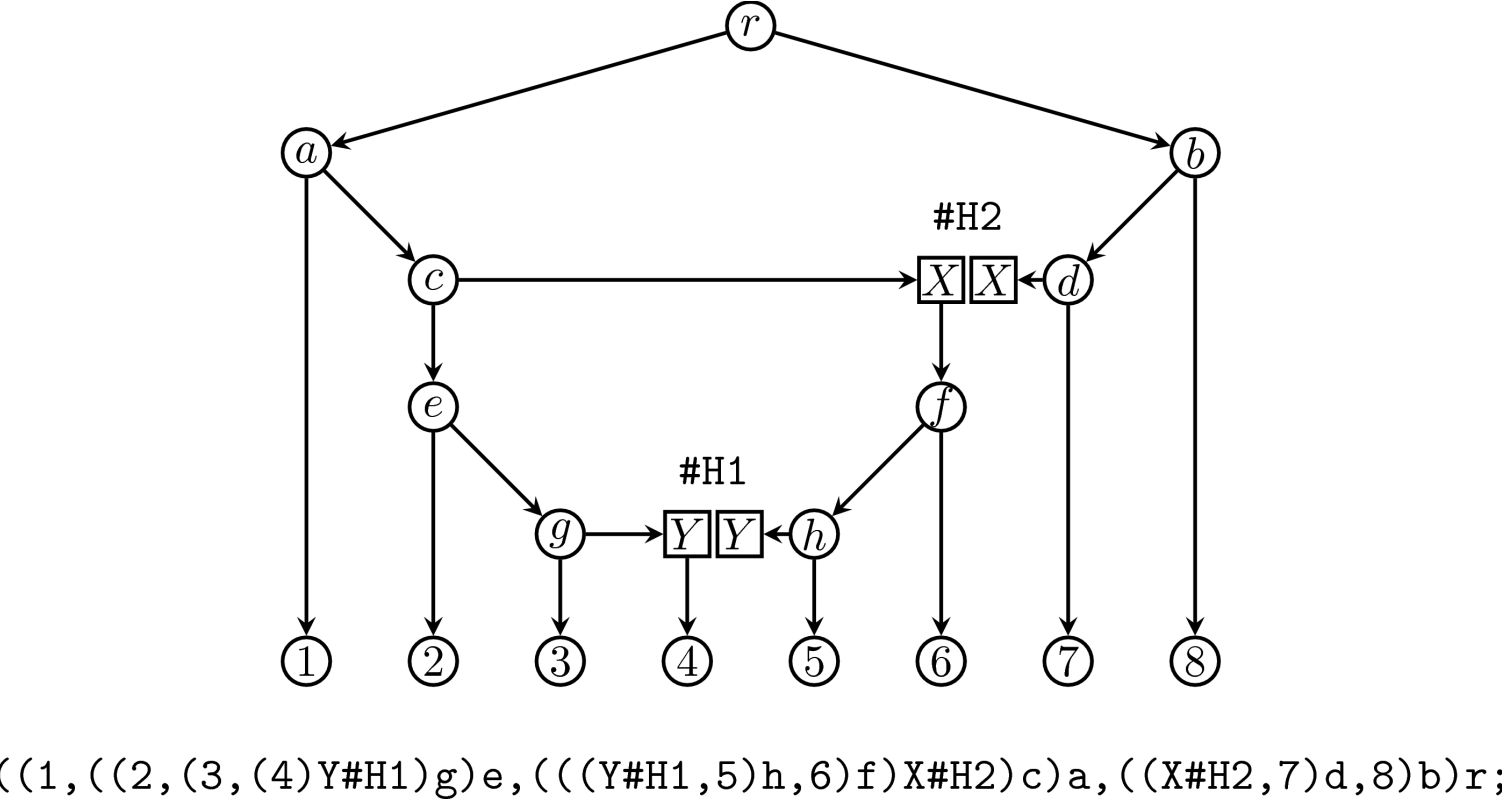
**Obtaining the extended Newick description of a phylogenetic network**. The phylogenetic network with two hybrid nodes of Figure 1 can be transformed into a phylogenetic tree with two replicated nodes (top), which can then be traversed in postorder to obtain the extended Newick description of the phylogenetic network (bottom).

A first proposal of a compact representation for phylogenetic networks can be found in the *NetGen *package for phylogenetic networks [11], where a phylogenetic network with *k *hybrid nodes is represented as a single phylogenetic tree in Newick format but with *k *repeated nodes. For example, the phylogenetic network with two hybrid nodes of Figure 1 is transformed by replicating each hybrid node as shown in Figure 2, and the resulting representation is the following Newick string:

• ((1, ((2, (3, (4)Y#H1)g)e, (((Y#H1, 5)h, 6)f)X#H2)c)a, ((X#H2, 7)d, 8)b)r;

The representation of a whole phylogenetic network as a single string facilitates phylogenetic data sharing and exchange, because the string can then be embedded in the text of email messages, a collection of these strings can be put together in a text file with one line for each string, etc. It also facilitates the use phylogenetic networks in computer programs because in programming languages such as C, C++ and Java, in scripting languages such as Perl and Python, and in text processing languages such as awk, sed and grep, for instance, the single string representing a phylogenetic network can be easily input to a program or script through a command line interface or a graphical user interface, or read as a single line from a text file.

**Figure 3:**
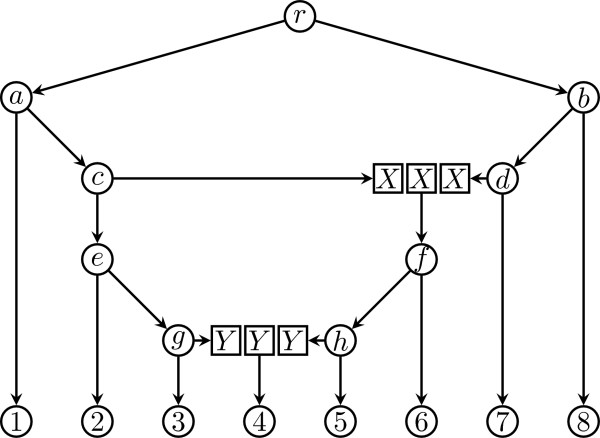
**Decomposing a phylogenetic network into a series of phylogenetic trees**. The phylogenetic network with two hybrid nodes of Figure 1 can be decomposed into three phylogenetic trees.

A second proposal of a compact representation for phylogenetic networks can be found in the *PhyloNet *package for phylogenetic trees and networks [12], where a phylogenetic network with *k *hybrid nodes is represented as a series of *k *+ 1 phylogenetic trees in Newick format. For example, the phylogenetic network with two hybrid nodes of Figure 1 is decomposed into three phylogenetic trees as shown in Figure 3, and the resulting representation is the following series of Newick strings:

• ((1, ((2, (3, Y)g)e, X)c)a, ((X, 7)d, 8)b)r;

• (((Y, 5)h, 6)f)X;

• (4)Y;

In the actual representation used in [12], however, the phylogenetic trees are represented by Newick strings (without the final semicolon, without root node label, and without any internal node labels) assigned either to the whole phylogenetic network or to the hybrid node, such as

• N = ((1, ((2, (3, Y)), X)), ((X, 7), 8))

• X = (((Y, 5), 6))

• Y = (4)

for the phylogenetic network with two hybrid nodes of Figure 1.

In any case, the representation of a phylogenetic network as a set of several strings makes it more difficult to share and exchange phylogenetic data, however, because it requires additional mark-up to properly keep the strings of different phylogenetic networks apart, especially when the strings of several phylogenetic networks are assembled together in a text file. Even in the case of a single phylogenetic network, though, the number of strings comprising the representation of the phylogenetic network is not made explicit in the representation and thus, additional mark-up is also needed in this case to indicate the end of the series of Newick strings. This second proposal results thus in a longer and more involved representation than the first proposal, using *k *more strings and 2*k *more symbols to represent a phylogenetic network with *k *hybrid nodes.

**Figure 4:**
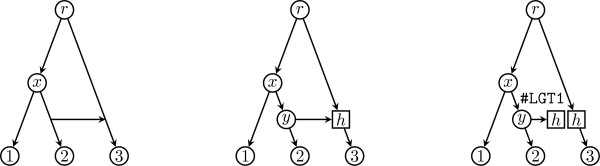
**Representing a lateral gene transfer event as a hybrid node**. Unique representation of a lateral gene transfer event (left) as a hybrid node in a phylogenetic network (right).

An additional drawback of this representation of a phylogenetic network as a set of several strings is the incomplete modeling of lateral gene transfer events, where the distinction between the reticulate edge and the other edge coming into the hybrid node is lost. The representation of a phylogenetic network is thus complemented in [12] with an explicit list of the lateral gene transfer arrows. For example, the lateral gene transfer event depicted in Figure 4 would be represented as follows.

• N = ((1, (2, H)), H)

• H = (3)

• 2 -> 3

Recently, in an open session at the Current Challenges and Problems in Phylogenetics workshop, held at the Isaac Newton Institute for Mathematical Sciences, Cambridge, UK in September 2007, several computational biologists gathered together and agreed upon an extended Newick format as a standard for the representation for phylogenetic networks. The meeting was followed by extensive discussion by email, with important contributions by Gabriel Cardona, Daniel Huson, Monique Morin, David Posada, and Gabriel Valiente.

The second proposal was discarded because of the practical issues of dealing with several Newick strings as the representation of a single phylogenetic network, and the first proposal was adopted with a few minor improvements. The agreed-upon standard was then included in the *Bio::PhyloNetwork *package for phylogenetic networks in Perl [13] and made available as part of the BioPerl bundle [14]. The extended Newick format is described in detail in the Results section.

Later on, however, the second proposal was claimed in [15] to be *the *extended Newick format for phylogenetic networks, although it was already discarded at the Current Challenges and Problems in Phylogenetics workshop for the reasons exposed above, and despite the fact that the extended Newick format for phylogenetic networks was already published [13]. This is, in our opinion, unfortunate for a series of reasons, the most important being that the publication under the same name and in the same journal of two standard formats for representing phylogenetic networks, will only add confusion and make it harder for biologists to share phylogenetic network data. This will be further addressed in the Discussion section.

Furthermore, there are a number of mistakes in the description of the second proposal as published in [15]. First, the description of a network with ℓ hybrid nodes is defined as a set of ℓ trees, but the description has ℓ + 1 trees instead. Second, in the procedure for decomposing a network into a set of trees, *k *new terminal nodes labeled *x*_*i *_are created for each hybrid node *u*_*i *_with *k *parent nodes *V*_*i*_, the edges *V*_*i *_× {*u*_*i*_} are removed and the edges *V*_*i *_× {*x*_*i*_} are added, but the latter is not well-defined because there are multiple nodes labeled *x*_*i *_while these edges are to be added from each parent in *V*_*i *_to only one new terminal node labeled *x*_*i*_. Third, the resulting set of trees is claimed to have the same terminal nodes as the network, but this is clearly false as new terminal nodes are introduced, multiple times indeed. Fourth, the resulting trees are also claimed to have disjoint sets of terminal nodes but this is also false, it does not even hold for the example in [[15], Figure 3].

## Results

The extended Newick format that we advocate as standard makes it possible to describe a whole phylogenetic network in linear form in a unique way, once the phylogenetic network is drawn or the ordering among children nodes is fixed. The extended Newick description of a phylogenetic network is a string of nested parentheses annotated with taxa names and possibly also with branch lengths or bootstrap values, similar to the Newick description of a phylogenetic tree but with additional mandatory tags to distinguish among the various hybrid nodes in the phylogenetic network.

The extended Newick description of a phylogenetic network can be obtained by first transforming the phylogenetic network into a phylogenetic tree with some replicated nodes, properly tagged according to the hybrid nodes they replicate, and then traversing the resulting phylogenetic tree in postorder as when obtaining the Newick description of a phylogenetic tree [6].

Given an order *H*_1_,..., *H*_*m *_on the hybrid nodes in a phylogenetic network, each hybrid node *H*_*i *_with *k *parents *u*_1_, *u*_2_,..., *u*_*k *_and ℓ children *v*_1_, *v*_2_,..., *v*_ℓ _is first split in *k *different nodes, the first such copy with parent *u*_1 _and children *v*_1_, *v*_2_,..., *v*_ℓ_, and the remaining *k *- 1 copies with a single parent *u*_2_,..., *u*_*k*_, respectively, and no children. Then, each tree node is labeled

[label] [:branch_length]

and the copies of each hybrid node *H*_*i *_are all labeled

[label]#[type]*i*[:branch_length]

where label is an optional string providing a labelling for the node; type is an optional string indicating if the hybrid node corresponds to a recombination (indicated by R), a hybridization (indicated by H) or a lateral gene transfer (indicated by LGT) event; the mandatory integer *i *identifies the hybrid node *H*_*i*_; and branch_length is an optional number giving the length of the branch from the parent to the copy of *H*_*i *_under consideration.

The unique representation of lateral gene transfer events as hybrid nodes, however, requires distinguishing the reticulate edge from the other edge coming into the hybrid node. This can be easily achieved by taking the target of the other edge as first replicate (the one that will carry the children of the hybrid node in the phylogenetic network) and the target of the reticulate edge as second replicate (the one that will become a terminal node) when splitting the hybrid node. For example, in the lateral gene transfer event depicted in Figure 4, represented by the extended Newick string ((1, (2, (3)h#LGT1)y)x, h#LGT1)r;, the reticulate edge *y *→ *h *makes the second copy of hybrid node *h *become a terminal node.

Conversely, given an extended Newick string, the corresponding phylogenetic network can be obtained by parsing the string into a phylogenetic tree with some replicated nodes and then merging all nodes with the same tag into a single node, which will become a hybrid node. For instance, merging the two nodes tagged Y#H1 into a single hybrid node, also tagged Y#H1, and merging the two nodes tagged X#H2 into a single hybrid node tagged X#H2 in the phylogenetic tree with repeated nodes of Figure 2, produces the phylogenetic network of Figure 1.

In a computer program or script, the special labeling of hybrid nodes can be used for instance to render tree nodes as circles, recombination or hybridization nodes as boxes, and lateral gene transfer nodes as arrows between edges.

## Discussion

The adoption of an extended Newick format as a standard for the representation of phylogenetic network is an important step towards the publication of explicit phylogenetic networks in peer-reviewed journals and their incorporation in a future database of published phylogenetic networks.

The representation of a phylogenetic network with *k *hybrid nodes as a single phylogenetic tree in Newick format but with *k *repeated nodes, first proposed in [11] and further developed in [13] and in this article, facilitates phylogenetic data sharing and exchange, and allows for the practical use of phylogenetic networks in computer programs and scripts. On the other hand, the representation of a phylogenetic network with *k *hybrid nodes as a series of *k *+ 1 phylogenetic trees in Newick format, recently proposed in [15], makes it more difficult to share and exchange phylogenetic data, and makes it much more difficult to deal with phylogenetic networks in computer programs and scripts. Besides, this representation is longer and more involved than the extended Newick format already published in [13], and it does not seem to add any new feature that make it deserve a new proposal.

The extended Newick format that we recommend as a standard for representing plylogenetic network is already available in the *Bio::PhyloNetwork *package for phylogenetic networks in Perl [13] as part of the BioPerl bundle [14], has been recently added to the SplitsTree tool for the reconstruction and visualization of implicit phylogenetic networks [16] and to the Dendroscope phylogenetic visualization tool [17], and will be supported in the next release of the TCS tool for phylogenetic network estimation using statistical parsimony [18].

Therefore, it will be in in the interest of community standards, and in the benefit of the bioinformatics community, that the authors adopt the standard we have already published in [13] and further described in this article.

## Authors' contributions

All authors prepared the manuscript, contributed to the discussion, and have approved the final manuscript.

## Response

By: Luay Nakhleh

Email: nakhleh@rice.edu

Dept. of Computer Science, Rice University, Houston, Texas, USA

While I am in complete agreement with the authors on the issue of standardization of formats, for all pertinent purposes, I think there are several issues that need to be settled before we declare or advocate a unique format for phylogenetic networks. I briefly discuss these issues next.

A phylogenetic tree represents the evolutionary history of a set of taxa.

While such a tree may come in different "forms" (rooted, unrooted, binary, multi-furcating, etc.), its definition is commonly agreed upon. On the other hand, a phylogenetic network is in fact an umbrella term that has been used by various computational biologists to mean different things. There are the "evolutionary networks", which are intended to model reticulate evolutionary relationships. There are the "splits graphs", which are intended to model any deviation from a tree model, whatever the cause may be. There are the "ancestral recombination graphs", which are intended to model evolutionary relationships at the population level, with recombination. And many more. All these models, though describe different scenarios and must be interpreted differently, are grouped under the term "phylogenetic networks". This issue is very significant to address before a "consensus" is reached and "mandated" about what the correct format is, if there is such a unique format, as the authors advocate. For example, I do not see how the splits graph version of phylogenetic networks can be meaningfully represented by any of the formats mentioned in the manuscript, including the one that my colleagues and I used in our recent paper [19]. Ancestral recombination graphs necessitate annotation of the nodes and edges of a network with information about the sites that mutated along an edge, and the sites between which recombination events may have occurred. Unlike the term 'phylogenetic tree' which is agreed upon already, the term 'phylogenetic network' currently encompasses an array of models that should not necessarily be treated the same.

I believe these issues need to be resolved before we address the issue of a standard representation for a diverse model such as phylogenetic networks.

The authors write in the Abstract that the extended Newick format they advocate is already supported by several phylogenetic tools. Later in the Discussion section, they noted that it is currently used only in their own Perl package [13], as well as in SplitsTree (and for this latter tool, it is not used for all purposes, as the authors indicate, which again, reflects the issue of different versions of phylogenetic networks).

These are only two tools of over twenty tools that have been developed so far for modeling different aspects of phylogenetic networks. The authors correctly note on Page 3 that "there is not yet, to the best of our knowledge, any effort to build and maintain a database of published phylogenetic networks." I concur with the authors on this. However, the reason for this, I believe, is not the lack of a standard format. It is because the area of phylogenetic networks is still in its infancy, and reconstruction methods are still too "primitive" to have their output be taken as a ground truth that requires storage in a database. The authors do not seem to acknowledge this fact, since they write in the first paragraph of the Background section that "phylogenetic networks have been extensively used in evolutionary studies." As I am a member of the phylogenetic networks community, I wish this were the case. However I disagree with the notion that phylogenetic networks have been extensively used. Further, there have been very few established data sets in the literature with evidence of reticulate evolution.

Publishing results on the extent of horizontal gene transfer, for example, does not mean that we can take these results as truths. There is significant debate in the evolutionary biology community about the extent of reticulate evolution, the role it plays, and the ways (and data required) to detect it.

The Newick format for representing trees was not devised for humans to read or write, but for automated parsing and production. Similarly, we do not expect a phylogenetic network representation for I/O operations would be for human reading. The authors criticize the representation that we adopted [19] stating that it "makes it more difficult to share and exchange phylogenetic data, however, because it requires additional mark-up to properly keep the strings of different phylogenetic networks apart, especially when the strings of several phylogenetic networks are assembled together in a text file." I would like to point out that phylogenetic tools, such as PAUP or MrBayes, in fact use a very rich language for I/O representation, called Nexus. This language allows for all sorts of annotations. Tools that use the Nexus format further customize it for their own purposes and functionalities. For example, PAUP has a 'paup' block; MrBayes has a 'mrbayes' block; and so on. I would not be surprised if specialized blocks for representing phylogenetic networks are added to the Nexus format.

The authors discuss on Page 5 their own meetings in 2007 and how they decided on a format and discarded of other formats. However, I was not part of that discussion. Further, PhyloNet [19] was made public for the first time in early 2006. The format we devised for phylogenetic networks (the evolutionary version of these) was adopted in PhyloNet before the mentioned meeting. The authors criticism of our discussion of "the extended Newick format" in our BMC Bioinformatics paper is unfounded. In their 2006 Bioinformatics paper, Morin and Moret [11] proposed a representation that we acknowledged in our BMC Bioinformatics paper. However, they did not use the term "extended Newick" or "eNewick", as far as I recall. Our reference to the extended Newick format was simply to the format we used in the PhyloNet tool. The authors missed that point, and thought that we meant to say that the format we presented was the one and only, which we never claimed or advocated (nor do we advocate).

Finally, the last paragraph on Page 5, in which the authors discuss typos in our manuscript, has nothing to do with the need for a standard representation of phylogenetic networks (which is the subject of the authors' paper), since these typos do not discredit or have anything to do with the appropriateness of the format we used in PhyloNet. Further, I had personally corresponded with one of the three authors about these specific typos and clarified to him what was meant. So, I am surprised to see them raising these points here, as if they were central to the issue of standard representation of phylogenetic networks. Nonetheless, I will answer them again:

About the l vs. l +1 trees in the representation of a network with l hybrid nodes, this is definitely a typo and our examples show that it has l +1 (not l). For each hybrid node ui, the point is that we attach each of its parents to a single leaf, yet all these leaves are labeled with xi. Figure 3 in our manuscript [19] clearly illustrates this. When we write that the set of trees have the same terminal nodes as the network, we meant that when these trees are restricted to the original set of leaves of the network before decomposition. About "the resulting trees have disjoin sets of terminal nodes", it is meant that no two trees have any leaf labels in common. This is also clear in Figure 3.

In summary, I do agree with the authors about the utility of a standard representation, but I am afraid that the different meanings the term 'phylogenetic network' is currently used to have, coupled with the fact that the entire discipline is still in its infancy warrant that we not restrict ourselves to one format and discard others.
